# Exploring body uneasiness in severe and enduring eating disorders: insights from clinical practice

**DOI:** 10.1186/s40337-024-01129-2

**Published:** 2024-10-18

**Authors:** Paolo Meneguzzo, Patrizia Todisco

**Affiliations:** 1 Department of Neuroscience, University of Padova, via Giustiniani 2, Padova, 35128 Italy; 2 Padova Neuroscience Center, University of Padova, Padova, Italy; 3 Eating Disorders Unit, Casa di Cura “Villa Margherita”, Arcugnano, VI Italy

**Keywords:** Body uneasiness, Eating disorders, Severe, Enduring, Compulsive self-monitoring

## Abstract

**Introduction:**

Body uneasiness is a central facet of body image disturbances observed in individuals with eating disorders (EDs). This study aimed to address gaps in understanding body uneasiness in severe and enduring eating disorders (SE-EDs) and explore variations in psychopathology between individuals with different durations of the disorder. We hypothesized that patients with SE-ED might develop habitual behaviors that contribute to ambivalence toward treatment and the persistence of symptoms.

**Methods:**

A sample of 360 ED patients was evaluated at the beginning and end of a specialized intensive rehabilitation program. All patients completed the Eating Disorder Examination Questionnaire (EDE-Q) and the Body Uneasiness Test (BUT). They were divided into two groups: SE-ED (> 7 years) and acute (aED, < 3 years) duration.

**Results:**

Compulsive self-monitoring showed a significant change between the start and end of treatment, differing between groups, with a larger change observed in SE-ED (*p* < 0.048). In SE-ED, it was associated with lower chances of dropout (*p* = 0.044), opposite to aED (*p* = 0.009). Treatment responses were primarily related to eating psychopathology, further highlighting differences between the two groups.

**Conclusions:**

This study underscores the possible presence of a habit in SE-ED and the importance of tailoring interventions to address unique needs based on the duration of the disorder. Furthermore, it highlights the need for further research to improve treatment outcomes in SE-EDs.

## Abstract (plain language summary)

This study looked at how people with severe and enduring eating disorders (SE-EDs) feel about their bodies and how it affects their treatment. We studied 360 people in a special treatment program. We found that people with SE-EDs were more likely to monitor their bodies a lot, and this made them less likely to stop treatment. This was different for people with short-term disorders. The way people felt about eating also affected how well they responded to treatment. This study shows that people with long-lasting eating disorders might have habits that affect their treatment, and we need to think about this when helping them. There is still a lot to learn about how to treat SE-EDs better.

## Introduction

Body uneasiness is a central facet of body image disturbances observed in individuals with eating disorders (EDs). It goes beyond mere dissatisfaction with body shape and weight, manifesting itself in behaviors such as avoidance, compulsive control actions, detachment from the body, and increased concerns about one’s physical appearance [1]. Furthermore, it has been associated with deficits in self-development, which impairs the ability to differentiate bodily needs from emotional experiences [2]. This uneasiness is shaped by a cognitive-affective disposition toward one’s body, correlated with more severe psychopathology and laying the groundwork for a pervasive sense of alienation from the body [1]. Unlike the concept of embodiment, this alienation from the body may become habitual for patients, serving as a defining aspect of their identity and a means of reclaiming a sense of lost self as suggested by previous literature on treatment response [3]. The loss of ownership of the sense of body is a key factor in understanding the negative body and its dissociation from it [4]. This phenomenon sheds light on potential explanations for deficits in embodiment ownership in ED patients [5, 6]. Additionally, body unease is prominent as a predictor of ED psychopathology and interpersonal challenges [7], as well as an indicator of embodiment disorders that exhibit greater resistance to treatment interventions [3, 8].

In this complex landscape of body image disturbance, a particularly challenging subgroup of eating disorders has emerged: Severe and Enduring Eating Disorder—SE-ED [9, 10]. SE-EDs represent a distinct clinical category characterized by the chronic and persistent nature of symptoms, often lasting for many years with minimal response to conventional treatment approaches [11, 12]. These disorders are not merely an extension of typical EDs but signify a deeper entrenchment of the illness within the individual’s psyche and daily life [13, 14]. SE-EDs challenge traditional treatment paradigms, which have historically focused on symptom reduction and targeting specific psychopathological features [12, 15]. Given the chronic nature of SE-EDs, there is a growing consensus in the literature that treatment must evolve to prioritize the overall quality of life for these patients, rather than simply aim for the remission of symptoms [16].

This paradigm shift is supported by compelling evidence indicating that concerns about body image, a key issue in ED, often persist even after treatment, particularly in those suffering from SE-EDs [10, 17]. These persistent concerns contribute to the complexity of recovery, as patients with SE-ED often experience profound ambivalence toward recovery. For them, eating disorder has become more than just an illness; it is a fundamental part of identity, deeply woven into their sense of self [18, 19]. This fusion complicates the therapeutic process, as the resolution can be perceived as a loss of self, rather than a restoration of health.

Therefore, understanding the role of body uneasiness in SE-ED is crucial for developing effective treatment strategies [20]. It is not just the presence of body image disturbance that poses a challenge, but how this disturbance is deeply rooted in the patient’s self-concept and daily experience [14]. Body image disturbance represents a reductionist approach in SE-EDs, and we should be aware that there are elements related to interoception, alexithymia, dissociation, embodiment, and shame [13]. As we move toward more holistic treatment approaches, recognizing the enduring nature of these concerns in SE-EDs can help clinicians tailor interventions that address the psychological and existential dimensions of these disorders, ultimately aiming to improve the overall quality of life of those affected [18].

 [10, 17]However, body uneasiness in SE-EDs remains less thoroughly evaluated. Our objectives are double. First, to investigate how body uneasiness and related psychopathological features differ between individuals with short-term and long-term experiences of the disorder. Second, to explore how these differences contribute to treatment outcomes and the persistence of symptoms. We hypothesize that patients with SE-ED may develop a distinct habit characterized by deep-seated body uneasiness, which could contribute to their ambivalence towards treatment and the chronic nature of their symptoms over time.

## Methods

This longitudinal study involved an initial evaluation at T0, at the beginning of treatment, using a specific questionnaire to address concerns about body image. Psychological scores derived from the questionnaire were used as predictors to assess their ability to predict dropout and response to treatment, which were evaluated at the end of the treatment period (T1).

### Participants

Patients seeking treatment and with a complete or subthreshold diagnosis of anorexia nervosa (AN) or bulimia nervosa (BN) according to the criteria of DSM-5 were consecutively enrolled in an inpatient Eating Disorders Unit of the Casa di Cura Villa Margherita (Arcugnano, Vicenza), between June 2014 and December 2023. For this study, people were enrolled if their duration of the disorder was shorter than 3 years (acute ED, aED) or longer than 7 years, according to the definition of SE-ED presented in the literature [21].

The sample included 360 cisgender females with an average age of 26.10 years (SD 10.36). Looking at the diagnosis, 246 people had a diagnosis of Anorexia Nervosa (68.33%); while 89 people (24.72%) were affected by Bulimia Nervosa (BN) and 36 participants (10.00%) by Other Specified Feeding or Eating Disorder (OSFED).

The study was approved by the Vicenza Ethics Committee as part of a clinical evaluation of ED patients hospitalized in the Unit and complies with the provisions of the Declaration of Helsinki. All patients included in the analysis were volunteers, accepted to participate in the study, and signed informed consent (or their parents if they were underage).

### Measure

All participants were clinically evaluated with weight, height, and the registration of the sex assigned at birth on the second day of hospitalization. Participants completed two validated questionnaires to record their eating psychopathology and body image difficulties twice: during the first week of hospitalization and the last week before discharge.

The Eating Disorders Examination Questionnaire (EDE-Q) is a self-report measure designed to assess key behaviors and thoughts related to eating disorders [22]. It consists of 28 items that cover various aspects of eating disorder behaviors and thoughts. Each item is rated on a 7-point Likert scale, with scores ranging from 0 to 6. The questionnaire yields four subscale scores: Dietary Restraint, Eating Concern, Weight Concern, and Shape Concern, and a global score reflecting the psychopathology of overall eating disorder psychopathology. In this study, the internal consistency was good with all subscales with a Cronbach alpha greater than 0.8.

Body Uneasiness Test (BUT) Part A was used to comprehensively assess body image disturbance through five distinct factors, using a 34-item, 6-point Likert scale [1]. Weight phobia (WP) delves into the fear of being or becoming fat, capturing anxieties surrounding weight gain and body size. Body Image Concerns (BIC) quantifies concerns related to physical appearance, encompassing a broad spectrum of concerns beyond weight. Avoidance (A) evaluates avoidance behaviors related to body image, revealing coping strategies to avoid distressing situations. Compulsive self-monitoring (CSM) measures the compulsive checking of physical appearance, reflecting obsessive tendencies in monitoring body size, shape, or weight. Depersonalization (D) explores feelings of detachment and estrangement from the body, uncovering disturbances in embodiment and self-identity. In this study, internal consistency was good with all subscales with a Cronbach’s alpha greater than 0.8.

### Treatment

The treatment program was designed according to widely accepted practice guidelines, integrating a multidisciplinary team approach that combined structured protocols with flexible patient-centered strategies [23, 24]. Each team member contributed to the development of customized interventions during weekly meetings. The specialized staff delivered a comprehensive array of services, including individual weekly psychotherapy sessions, group therapies, nutritional guidance, nursing care, meal planning and supervision, family therapy, psycho-educational groups, and, when necessary, psychopharmacological treatments. Based on cognitive behavioral therapies, the treatment incorporated personalized interventions informed by third-wave CBT techniques such as mindfulness and sensorimotor therapy, which were supplementary to standard care. This approach prioritized the individual needs, symptoms, and history of each patient.

### Statistical plan

Differences at baseline (T0) were assessed using the Mann-Whitney test for variables exhibiting nonparametric distributions, while the chi-square test was used for categorical variables. Changes between T0 and T1 were evaluated using the Wilcoxon test for paired samples. We evaluated the interaction between effect of the Time (changes between T0 and T1) × Group (aED and SE-ED) using General Linear Models for repeated measures, examining how changes over time interacted with the duration of the disorders.

Finally, logistic regressions were employed to assess the predictive value of the BUT for dropout and treatment responses in both aED and SE-ED. Dropout was defined as the interruption of hospitalization against medical advice prior to the completion of the rehabilitation project. The response to treatment, measured at the end of hospitalization (T1), was defined as achieving an EDE-Q global score < 2.3, consistent with the mean + 1 standard deviation of the score of the normative Italian population, a methodology recommended in previous literature [25]. Ages, BMI, and psychological scores were examined as independent variables in separate multiple logistic regression analyzes for aED and SE-ED, considering dropout and treatment responses as dependent variables [25]. Statistical analyzes were performed using SPSS version 25, with a significance level set at α = 0.05.

## Results

### Clinical evaluation

Out of the total 360 participants in the study, 180 individuals (50.00% of the sample) were classified into the SE-ED group. Table 1 presents the group comparisons, revealing that the SE-ED cohort tended to be older and had earlier onset of the disorder. Regarding psychopathological characteristics, only the CSM subscale exhibited significant differences, showing lower scores in the SE-ED group.

**Table 1:** Demographic and clinical features at T0

	aED (*n* = 180)	SE-ED (*n* = 180)	Z	*p*
Age, years	19.48 (6.04)	32.62 (9.56)	-14.103	< 0.001
BMI, kg/m^2^	17.60 (5.14)	18.19 (4.91)	-1.877	0.061
Age of onset, years	17.65 (6.08)	16.41 (5.90)	-3.168	0.002
Duration of the disorder, years	1.83 (0.93)	16.21 (8.08)	-16.754	< 0.001
Diagnosis	AN = 130 (72.22%) BN = 39 (21.67%) OSFED = 11 (6.11%)	AN = 110 (61.11%) BN = 48 (26.67%) OSFED = 22 (12.22%)	5.630 §	0.060
EDE-Q				
Restraint	3.71 (1.71)	3.53 (1.92)	-0.635	0.525
Eating Concern	3.24 (1.45)	3.45 (1.61)	-1.475	0.140
Shape Concern	4.06 (1.59)	4.09 (1.60)	-0.236	0.814
Weight Concern	4.58 (1.42)	4.63 (1.42)	-0.522	0.601
Global score	3.90 (1.33)	3.92 (1.36)	-0.426	0.670
BUT				
WP	3.42 (1.16)	3.30 (1.16)	-1.187	0.235
BIC	3.23 (1.22)	3.15 (1.17)	-0.799	0.424
A	1.41 (0.93)	1.64 (1.21)	-1.133	0.257
CSM	2.90 (1.22)	2.42 (1.33)	-3.714	< 0.001
D	1.96 (1.00)	2.08 (1.21)	-0.819	0.413
GSI	2.95 (1.04)	2.85 (1.05)	-0.866	0.387

§: chi-square analysis; aED: acute eating disorder; SE-ED: severe enduring ED; EDE-Q: eating disorder examination questionnaire; BUT: body useanisness test; WP: weight phobia; BIC: body image concerns; A: avoidance; CSM: compulsive self-monitoring; D: depersonalization; GSI: global severity index

### Longitudinal evaluations

In our longitudinal evaluation of the treatment, we observed that 292 of 360 participants (81.11%) completed the treatment protocol, with no significant variances between the groups: aED *n* = 143 (out of 180, 79.44%) and SE-ED *n* = 149 (out of 180, 82.78%), χ2 (1) = 0.213, *p* = 0.644. Among these 292 individuals, 122 (41.8%) reported a notable decrease in scores on the EDE-Q questionnaire, indicating a positive response to treatment. There was no significant contrast between the aED and SE-ED groups: aED *n* = 66 (46.2%), SE-ED *n* = 56 (37.6%), χ2 (1) = 2.203, *p* = 0.138.

Examining the changes between T0 and T1, only the CSM subscale showed a significantly larger reduction in SE-ED compared to aED. See Table 2 for more details.

**Table 2:** T1 data and analysis of changes from T0 to T1 for completers. See table 1 for T1-values

	aED (*n* = 143)	Z *p*		SE-ED (*n* = 149)	Z *p*		Time x Group *p*
	T1	Changes from T0 to T1		T1	Changes from T0 to T1		
EDE-Q							
Restraint	1.32 (1.69)	9.870 < 0.001		1.49 (1.27)	8.915 < 0.001		1.275 0.260
Eating Concern	2.05 (1.34)	7.603 < 0.001		2.07 (1.49)	7.408 < 0.001		2.776 0.097
Shape Concern	3.91 (1.71)	4.056 < 0.001		3.91 (1.80)	5.288 < 0.001		0.063 0.801
Weight Concern	3.05 (1.70)	6.245 < 0.001		3.19 (1.76)	5.824 < 0.001		0.796 0.373
Global score	2.58 (1.33)	8.845 < 0.001		2.66 (1.39)	8.573 < 0.001		0.142 0.707
BUT							
WP	3.09 (1.27)	2.510 0.012		3.03 (1.25)	3.048 0.002		0.028 0.866
BIC	2.77 (1.29)	1.860 0.046		2.78 (1.37)	3.547 < 0.001		1.235 0.267
A	1.03 (1.03)	3.424 < 0.001		1.34 (1.07)	3.402 < 0.001		0.552 0.458
CSM	2.61 (1.28)	1.564 0.118		2.05 (1.27)	4.010 < 0.001		3.835 0.048
D	1.66 (1.04)	2.495 0.013		1.79 (1.16)	2.615 0.009		0.585 0.445
GSI	2.53 (1.14)	2.935 0.003		2.47 (1.17)	4.427 < 0.001		0.693 0.406

Time × Group column reported the F-values and represents the interaction between changes from T0 to T1 (time) and the duration of the disorder (group aED or SE-ED). aED: acute eating disorder; SE-ED: severe enduring ED; EDE-Q: eating disorder examination questionnaire; BUT: body useanisness test; WP: weight phobia; BIC: body image concerns; A: avoidance; CSM: compulsive self-monitoring; D: depersonalization; GSI: global severity index

### Logistic regressions

Logistic regression analyzes revealed opposite odds regarding dropout from treatment for aED and SE-ED. Specifically, individuals with aED exhibited higher odds of dropping out with higher scores of compulsive self-monitoring (*p* = 0.009), while ED individuals with SE-ED had lower odds of dropping out with higher scores of compulsive self-monitoring (*p* = 0.044).

In terms of response to treatment, the logistic regressions indicated that individuals with aED reported lower odds of response with higher eating restraint (*p* = 0.025). On the contrary, people with SE-ED had a higher probability of response with higher scores on eating concerns (*p* = 0.033) and a lower probability with higher scores on shape concerns (*p* = 0.027). See Table 3 for details.

**Table 3:** Logistic regression analyzes

	aED				SE-ED		
	B	OR	95% CI for OR		B	OR	95% CI for OR
Drop out							
Age	0.178	1.195	0.794–1.799		-0.036	0.964	0.916–1.014
Age of onset	-0.147	0.863	0.574–1.300		-0.013	0.987	0.910–1.070
Restraint	-0.014	0.986	0.732–1.328		-0.020	0.980	0.722–1.244
Eating Concern	0.008	1.008	0.697–1.459		-0.218	0.804	0.589–1.098
Shape Concern	0.444	1.559	0.826–2.941		-0.091	0.913	0.496–1.683
Weight Concern	-0.387	0.679	0.421–1.096		0.276	1.318	0.774–2.245
WP	-0.568	0.566	0.293–1.095		0.360	1.433	0.749–2.586
BIC	0.028	1.029	0.588–1.802		-0.319	0.727	0.405–1.304
A	0.534	1.705	0.975–2.982		-0.164	0.849	0.517–1.392
CSM	**0.725**	**2.065**	**1.201–3.551**		**-0.489**	**0.613**	**0.399–0.942**
D	0.090	1.094	0.603–1.983		0.307	1.360	0.802–2.305
Treatment response							
Age	-0.283	0.753	0.515–1.102		0.040	1.041	0.995–1.089
of onset	0.262	1.300	0.890–1.899		0.027	1.027	0.960–1.099
Restraint	**-0.310**	**0.733**	**0.559–0.961**		-0.018	0.982	0.787–1.226
Eating Concern	-0.150	0.861	0.620–1.195		**0.323**	**1.381**	**1.024–1.863**
Shape Concern	-0.155	0.856	0.519–1.413		**-0.624**	**0.536**	**0.305–0.940**
Weight Concern	-0.017	0.984	0.657–1.474		-0.055	0.947	0.606–1.478
WP	0.427	1.532	0.854–2.749		-0.220	0.803	0.464–1.388
BIC	-0.335	0.715	0.441–1.161		0.039	1.039	0.615–1.758
A	0.231	1.260	0.749–2.119		-0.061	0.940	0.611–1.448
CSM	-0.238	0.788	0.491–1.266		0.273	1.313	0.783–1.975
D	-0.357	0.700	0.406–1.206		0.131	1.140	0.729–1.782

aED: acute eating disorder; SE-ED: severe enduring ED; EDE-Q: eating disorder examination questionnaire; BUT: body useanisness test; WP: weight phobia; BIC: body image concerns; A: avoidance; CSM: compulsive self-monitoring; D: depersonalization

## Discussion

This study aimed to address the gaps in understanding body uneasiness in SEED and explore how psychopathology can vary between individuals with different durations of the disorder. Furthermore, we sought to investigate whether SE-ED patients can develop a habit, contributing to their ambivalence toward treatment and the persistence of symptoms over the years.

Our findings contribute to the existing literature by providing deeper information on the psychopathological profiles of people with SE-ED compared to those with shorter-term experiences of the condition. In particular, the SE-ED group was significantly older, reflecting the long-lasting nature of these disorders and highlighting the need for age-specific treatments tailored to individuals with a long history of these conditions.

The elevated levels of CSM observed in the SE-ED group are particularly notable. CSM encompasses behaviors indicative of increased body dissatisfaction and obsessive tendencies, which may contribute to the chronicity of the disease in this group. These findings align with our hypothesis that people with SE-EDs can develop a habit characterized by established disordered eating patterns, body control, and ambivalence towards treatment. This is in line with studies that demonstrate the worst outcomes in patients with obsessive-compulsive ED [26] and the genetic overlap between ED and obsessive-compulsive disorders [27]. Furthermore, longitudinal analyzes showed that CSM had opposite roles in the dropout of people with a long-lasting or short-lasting disorder. This suggests potential challenges in adherence to treatment among people with recent-onset disorders when they have obsessive-compulsive characteristics exhibited by their compulsive behavior of body monitoring. On the contrary, people with SE-ED may be more inclined to remain in treatment despite the chronicity of their disease with higher levels of CSM, which probably represents obsessive-compulsive behavior that decreases anxiety and stress associated with treatment and the necessary change in their routine lifestyle. This aspect could also be related to personal history, which emphasizes the idea of a specific role for aversive events in the clinical presentation of ED patients. In particular, low parental care has been associated with weight phobia, avoidance, and depersonalization, while parental overprotection predicts the presence of compulsive self-monitoring [2].

Finally, our analysis of participants’ response rates to inpatient treatment revealed no significant differences between groups in terms of improvement in eating psychopathology. A similar response rate is consistent with the limited literature suggesting that eating psychopathology can improve even after a long period of illness, showing similar outcomes to those in patients with more recent onset [28, 29]. This improvement has been attributed to various non-specific factors affecting eating psychopathology, such as self-esteem, clinical comorbidities (e.g., personality disorders), psychosocial elements, and age of onset [11, 30]. These findings underscore the need for new treatment approaches and offer hope for further improvement even after a long history of disease [15, 31], calling for specific resources and options [32]. Furthermore, we identified a specific characteristic that may differentiate aED from SE-ED, as CSM showed a greater improvement in the SE-ED group. Compulsive self-monitoring has been associated with embodiment difficulties, concerns about body image, dysfunctional exercise, and parental bonding, highlighting its specific relevance in eating disorders [2, 5, 6, 33]. Our data showed an increased dropout rate in individuals with higher CSM, but a reduced dropout rate in SE-ED patients with higher CSM. These findings suggest that compulsive self-monitoring can become an automatic, habitual behavior in patients with SE-ED, leading to progressively ingrained body monitoring as the disorder persists, as reported in a qualitative study [17]. In contrast, in aED, this monitoring may be more complicated, as it can be heavily influenced by biased thoughts and cognitive distortions related to the disorder [34–36]. This may also suggest that in SE-ED, compulsive self-monitoring could serve as a coping mechanism that becomes more ingrained over time, helping patients maintain a sense of control in a context of lost self- [14]. This ingrained behavior could explain the reduced dropout rates in patients with SE-ED, as they may find stability and comfort in this routine, allowing a more consistent engagement with treatment and controlled changes [37]. However, the absence of a follow-up evaluation prevents further speculation on the long-term implications of these findings.

However, our analysis revealed differential patterns in response to treatment between SE-ED and recent-onset eating disorder groups. Specifically, logistic regression analysis indicated that higher levels of restraint could hinder treatment efficacy, possibly due to the ‘honeymoon’ phase often observed in the early stages. On the contrary, eating concerns appeared to be associated with the effectiveness of treatment in people with long-standing eating disorders. This supports the hypothesis that restrictive behaviors in SE-ED represent established habits [38, 39], while shape concerns, fundamental to the disorder and difficult to address in treatment, can exacerbate outcomes.

The study benefits from a large sample size, improving statistical power and reliability. Conducting the study in a specialized treatment center ensures standardized care, minimizes confounders, and improves internal validity. However, reliance on self-report questionnaires introduces potential bias, and monocentric data limits generalizability. Another specific limitation of the study is that it includes only cisgender women, thus excluding males and individuals of different genders. A specific limitation of the study is the lack of information on previous treatments. Although all participants had undergone at least one specialized treatment before enrollment, the specific effects of the number and types of treatments could not be evaluated. Future research should replicate findings in diverse settings and with diverse populations, to improve external validity.

## Conclusions

This study provides important information on SE-ED, highlighting the impact of the duration of the disorder on psychopathological profiles and treatment responses. Our findings suggest that SE-ED patients can develop habitual behaviors, such as compulsive self-monitoring, which could contribute to the chronicity and persistence of symptoms. Despite the chronic nature of SE-ED, we observed a potential improvement in eating psychopathology, comparable to that of recent-onset cases. However, the differential response patterns between SE-ED and acute cases underscore the need for tailored treatment approaches. Restrictive behaviors in SE-ED may represent deeply ingrained habits, while shape concerns remain a core challenge.

Future research should further explore these findings to better understand and address the unique needs of SE-ED patients, to improve recovery outcomes. Understanding habitual behaviors and other psychopathological nuances of long-standing eating disorders is crucial to improve recovery rates and offering hope to those affected by these complex conditions.

## Data Availability

No datasets were generated or analysed during the current study.

## Data Sharing and Data Accessibility

Data supporting the findings of this study are available from the corresponding author on a reasonable request.
